# Pregnancy‐Associated Isolated Fallopian Tube Torsion With a True Knot Managed by Laparoscopic Salpingectomy: A Case Report

**DOI:** 10.1155/crog/8852614

**Published:** 2026-09-11

**Authors:** Ahmed Abdeltawab

**Affiliations:** ^1^ Obstetrics and Gynaecology Department, Farwaniya Hospital, Farwaniya, Kuwait; ^2^ Obstetrics and Gynaecology Department, Al-Azhar University, New Damietta, Egypt, azhar.edu.eg

**Keywords:** acute abdomen, isolated fallopian tube torsion, laparoscopy, pregnancy, salpingectomy, true knot

## Abstract

Isolated torsion of the fallopian tube is a rare cause of acute abdomen in women of reproductive age, with an estimated annual incidence of approximately one per 1.5 million women. Its occurrence during pregnancy is rarer still, with fewer than 50 cases published. We present a 25‐year‐old primigravida at 27+6 weeks of gestation who was admitted to Farwaniya Hospital, Kuwait, with persistent right iliac fossa pain refractory to conservative management, associated with nausea and dysuria, on a background of recurrent urinary tract infection. Laboratory investigations showed leucocytosis and mild anaemia. Transabdominal ultrasound with colour Doppler identified a small simple right adnexal cystic lesion with preserved ovarian vascularity, and pelvic magnetic resonance imaging demonstrated an encysted fluid collection in the right iliac fossa of probable adnexal origin, together with a nondistended, normal‐appearing appendix showing no features of inflammation. Following failure of conservative treatment and after counselling of the patient and her husband, diagnostic laparoscopy was performed. The right fallopian tube was gangrenous, twisted repeatedly upon itself and configured as a true knot, and salpingectomy was performed. Both ovaries and the appendix were macroscopically normal. Histopathology confirmed haemorrhagic infarction with epithelial necrosis of the tubal mucosa. The postoperative course was uneventful and the pregnancy continued to term, with spontaneous vaginal delivery of a healthy female neonate at 40+3 weeks of gestation. This case illustrates the diagnostic difficulty of isolated tubal torsion in pregnancy, in which urinary symptoms and non‐specific imaging readily divert attention towards pyelonephritis, and supports laparoscopy as a safe and definitive diagnostic and therapeutic option in the second trimester. The intraoperative finding of a true knot is unusual and is documented here with annotated operative imaging.

## 1. Introduction

Isolated torsion of the fallopian tube (IFTT), in which the tube twists upon its own axis while the ipsilateral ovary is spared, was first described by Bland‐Sutton in 1890 [1]. It is rare: The only population‐based estimate derives from a Danish survey of surgical and gynaecological departments conducted between 1957 and 1967, which suggested an annual incidence of approximately one per 1.5 million women [2]. Its occurrence during pregnancy is rarer still, with fewer than 50 cases published to date [3]; 19 were collected in a review spanning 1936 to 2009 [4], and a further 10 were identified in a systematic review of the following decade [5]. Recognised predisposing factors include hydrosalpinx, paraovarian or paratubal cysts, pelvic inflammatory disease, previous pelvic surgery with adhesions and mechanical factors such as an elongated mesosalpinx or the enlarging gravid uterus [3, 6, 7]; in many cases no predisposing factor is identified. Torsion predominates on the right, reported in more than 90% of cases in one review and attributed to the protective effect of the sigmoid colon, which limits mobility of the left adnexa, whereas the right adnexa lies alongside the more mobile caecum and ileum [3]; an equal distribution between sides has, however, been described in an adolescent series [8]. The clinical presentation is non‐specific and readily mimics appendicitis, ovarian torsion and renal colic [9]. Ultrasound with colour Doppler is rarely conclusive: Preserved flow does not exclude tubal torsion, because the tube and ovary receive a dual blood supply from both the ovarian and uterine arteries, and the whirlpool sign of a twisted vascular pedicle, seen between a morphologically normal ovary and the uterus, is the most useful sonographic clue [10]. In a 12‐year series of 17 surgically confirmed cases, not one was diagnosed preoperatively [11]. Magnetic resonance imaging (MRI) is valuable in pregnancy chiefly for excluding appendicitis and characterising adnexal pathology rather than for confirming torsion [10, 12]. Definitive diagnosis therefore rests on surgical exploration, and laparoscopy is recognised as safe during pregnancy when clinically indicated [13]. Delay in diagnosis commonly leads to necrosis of the tube and salpingectomy, with implications for future fertility. We present a case of IFTT with a true knot and complete gangrene complicating pregnancy at 27+6 weeks, managed successfully by laparoscopic salpingectomy with a favourable maternal and fetal outcome.

## 2. Case Presentation

A 25‐year‐old Egyptian primigravida (Gravida 1, Para 0) at 27+6 weeks of gestation presented to the Obstetrics and Gynaecology Emergency at Farwaniya Hospital, Kuwait, with a 2‐day history of sudden‐onset severe right iliac fossa pain radiating to the groin and right renal angle, associated with nausea and vomiting. She denied fever, vaginal bleeding or loss of amniotic fluid. Her past surgical history included haemorrhoidectomy and tympanoplasty. She reported a history of recurrent urinary tract infections (UTIs) both before and during pregnancy, with the most recent episode treated with nitrofurantoin, resulting in a negative urine culture. She had no known drug allergies and was on routine prenatal vitamins.

On initial assessment, she appeared in pain and distressed. Vital signs were temperature 36.9°C, pulse 86 beats per minute, blood pressure 95/70 mmHg and oxygen saturation 100% on room air. Abdominal examination revealed a soft abdomen with mild right renal angle and right iliac fossa tenderness, without rebound or guarding. Fundal height corresponded to gestational age. Fetal heart sounds were positive, and a viable fetus was confirmed on obstetric ultrasound. Pain did not respond to intravenous hyoscine butylbromide (20 mg) and paracetamol (1 g).

Laboratory investigations on admission showed a haemoglobin of 9.7 g/dL, falling to 8.7 g/dL on reassessment (pregnancy reference range 11.0–14.0 g/dL); a white blood cell count of 15.0–16.4 × 109/L with neutrophilia (pregnancy reference range 6.0–16.0 × 109/L); and a platelet count of 258 × 109/L (150–400 × 109/L). C‐reactive protein (CRP) was 10 mg/L (reference range < 5 mg/L) and procalcitonin (PCT) 0.091 *μ*g/L (reference range<0.5 *μ*g/L). Renal and liver function tests were mildly deranged. Urine cultures showed mixed growth or no growth. Serum amylase 76 U/L (28–100 U/L) and lipase 44 U/L (13–60 U/L) were both within normal limits, and lactate was 0.88 mmol/L (0.5–2.2 mmol/L). All reference intervals are those of the reporting laboratory at Farwaniya Hospital, adjusted for pregnancy where applicable.

**Figure 1:**
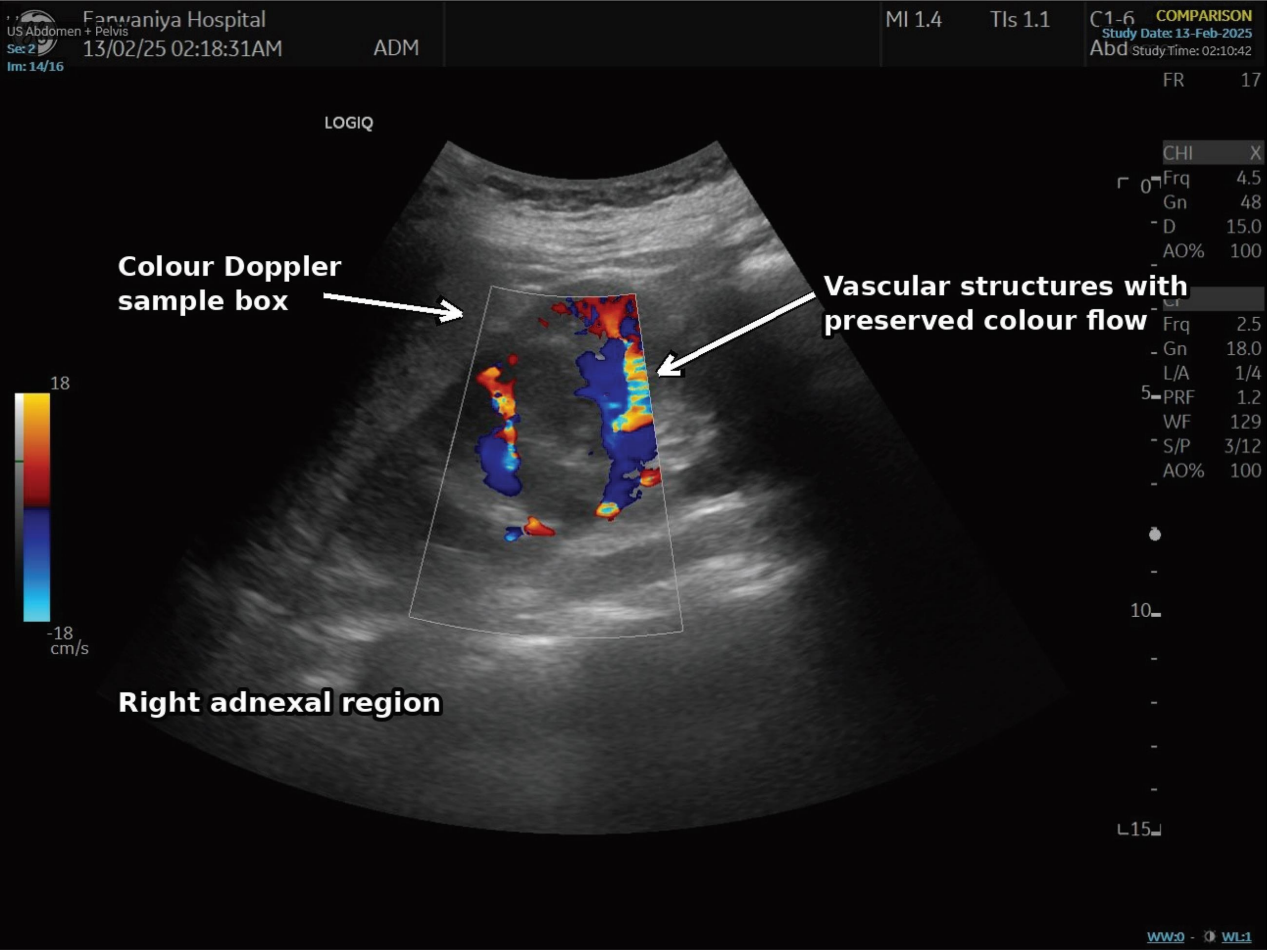
Transabdominal colour Doppler ultrasound of the right adnexal region. The colour Doppler sample box and vascular structures showing preserved colour flow are annotated. The small simple right adnexal cystic lesion of approximately 2.5–3 cm described in the contemporaneous radiology report is not resolved on this archived static image; the full sonographic findings are reported in the text. Preserved colour flow does not exclude tubal torsion because of the dual blood supply of the fallopian tube.

**Figure 2:**
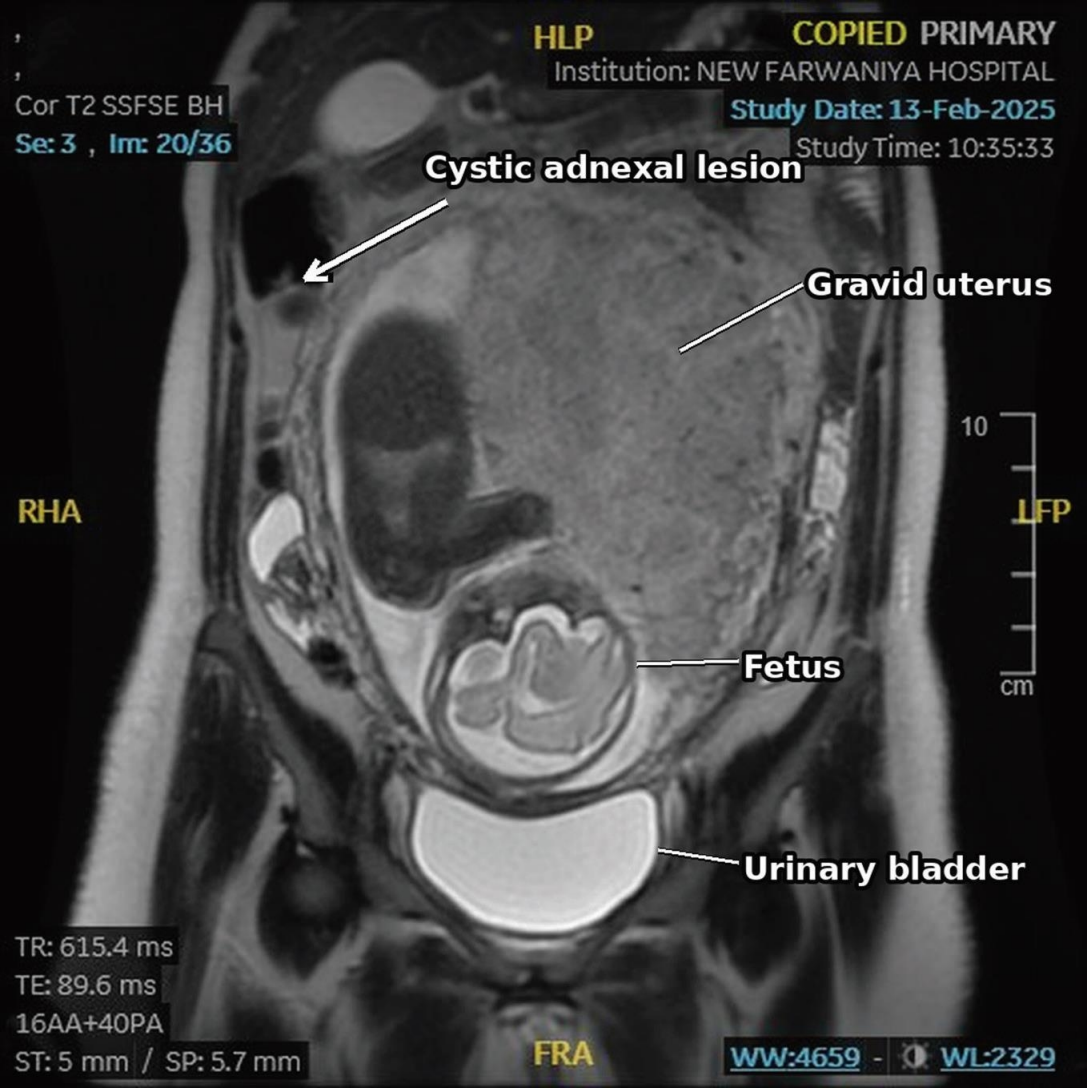
Pelvic MRI, coronal T2‐weighted single‐shot fast spin‐echo sequence. The white arrow indicates the encysted adnexal lesion in the right iliac fossa, reported on the contemporaneous radiology examination to measure 1.9 × 3.8 cm and to be inseparable from the right adnexa. The gravid uterus, the fetus and the urinary bladder are labelled. “RHA” on the left of the image denotes the patient′s right side.

**Figure 3:**
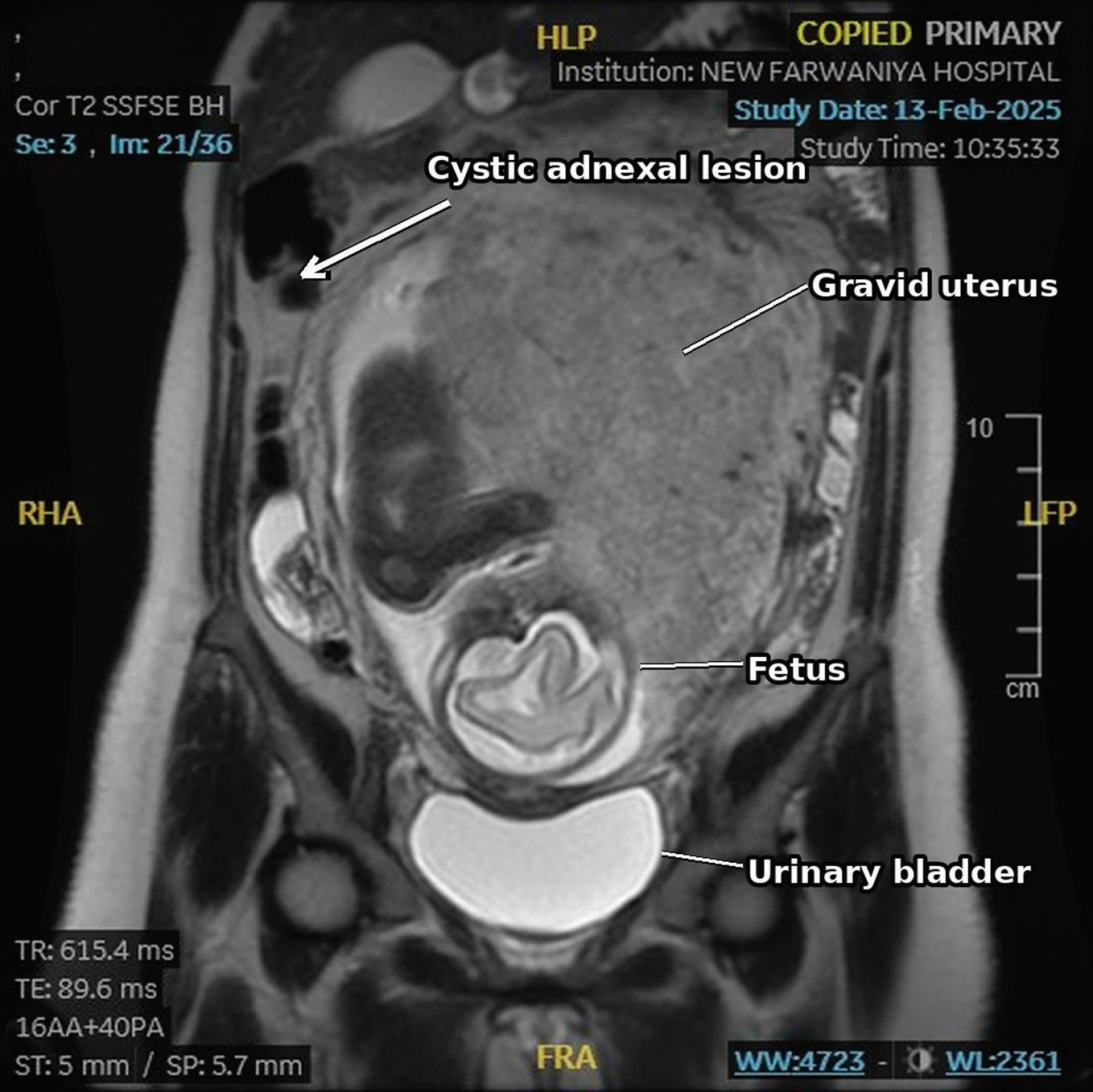
Pelvic MRI, adjacent coronal T2‐weighted slice from the same acquisition, confirming the adnexal lesion (white arrow) on two contiguous sections. Annotations as in Figure 2.

Abdominal and pelvic ultrasound with colour Doppler demonstrated a small simple right adnexal cystic lesion measuring approximately 2.5–3 cm, with preserved ovarian vascularity and no sonographic evidence of ovarian torsion (Figure 1). The appendix could not be visualised. Pelvic MRI, performed on the 12th day of admission, reported an encysted fluid collection in the right iliac fossa measuring 1.9 × 3.8 cm, inseparable from the right adnexa, displaying high T2 and low T1 signal, with minimal adjacent free fluid (Figures 2 and 3). A nondistended tubular structure was noted adjacent to the ascending colon and was interpreted as a collapsed appendix—that is, a normal, nondistended appendix rather than a decompressed appendix following perforation. There was no appendiceal wall thickening, periappendiceal fat stranding, free intraperitoneal air, extraluminal fluid collection or abscess to suggest either acute appendicitis or appendiceal perforation, and a macroscopically normal appendix was subsequently confirmed at laparoscopy. The MRI conclusion favoured an adnexal origin for the collection.

The patient was initially managed conservatively with intravenous antibiotics (ceftriaxone 2 g once daily), analgesics and intravenous fluids. Serial clinical assessments showed persistent severe right‐sided pain unresponsive to opioid analgesia (pethidine 50–100 mg). Surgical consultation was obtained and the possibility of appendicitis was considered but felt unlikely given the imaging findings. Given the failure of conservative management and persistent uncontrolled pain, a decision was made to proceed to diagnostic laparoscopy after multidisciplinary discussion. The patient and her husband were thoroughly counselled regarding the diagnosis, surgical risks including the risk of miscarriage, the possibility of salpingectomy and its implications for future fertility. A neonatology team was informed and on standby. Written informed consent was obtained. Dexamethasone was administered for fetal lung maturity. Diagnostic laparoscopy was undertaken on the thirteenth day of admission, approximately 2 weeks after the onset of pain.

**Figure 4:**
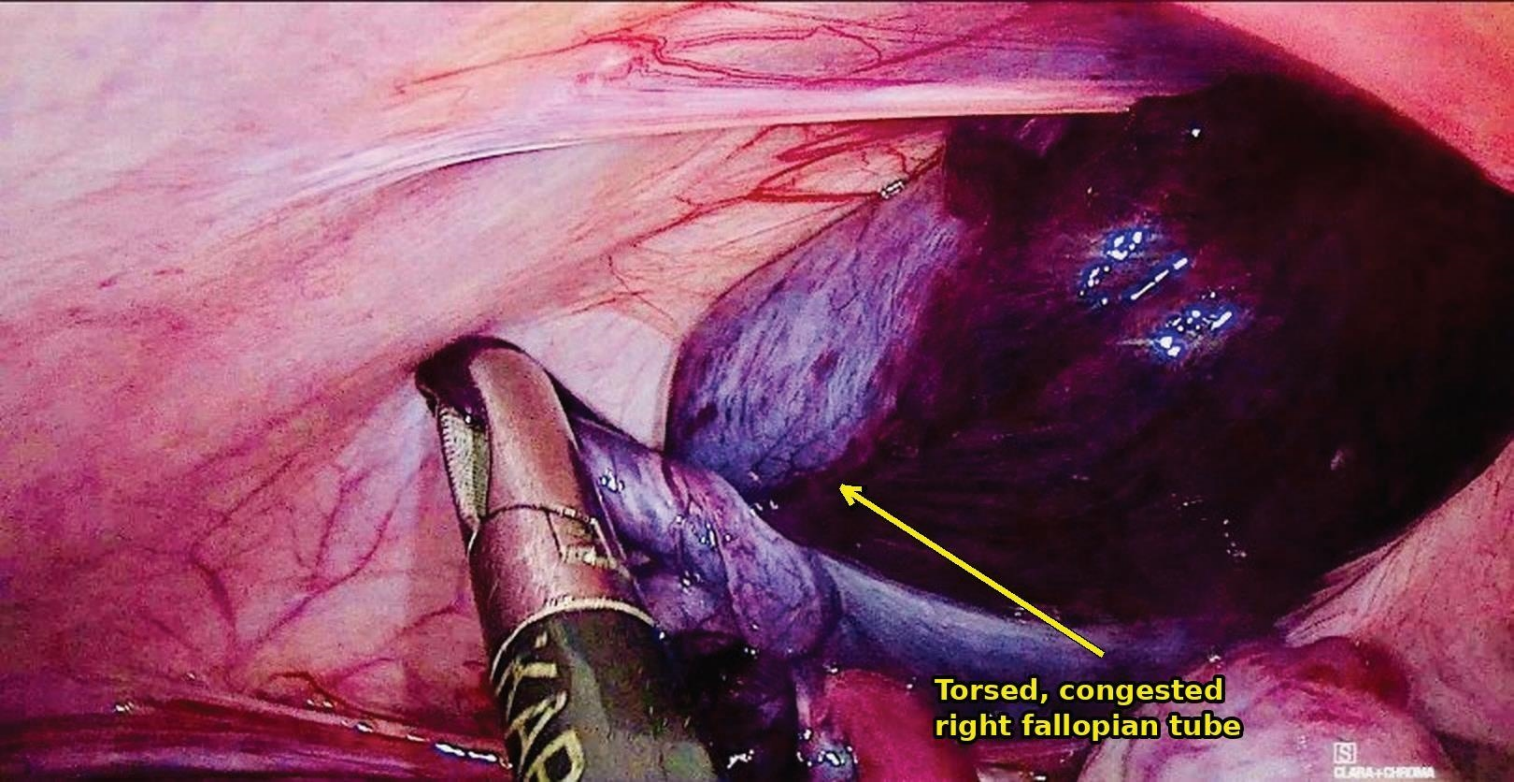
Laparoscopic view showing the congested and twisted right fallopian tube with multiple torsions and early necrotic changes. The yellow arrow indicates the torsed, congested tube.

**Figure 5:**
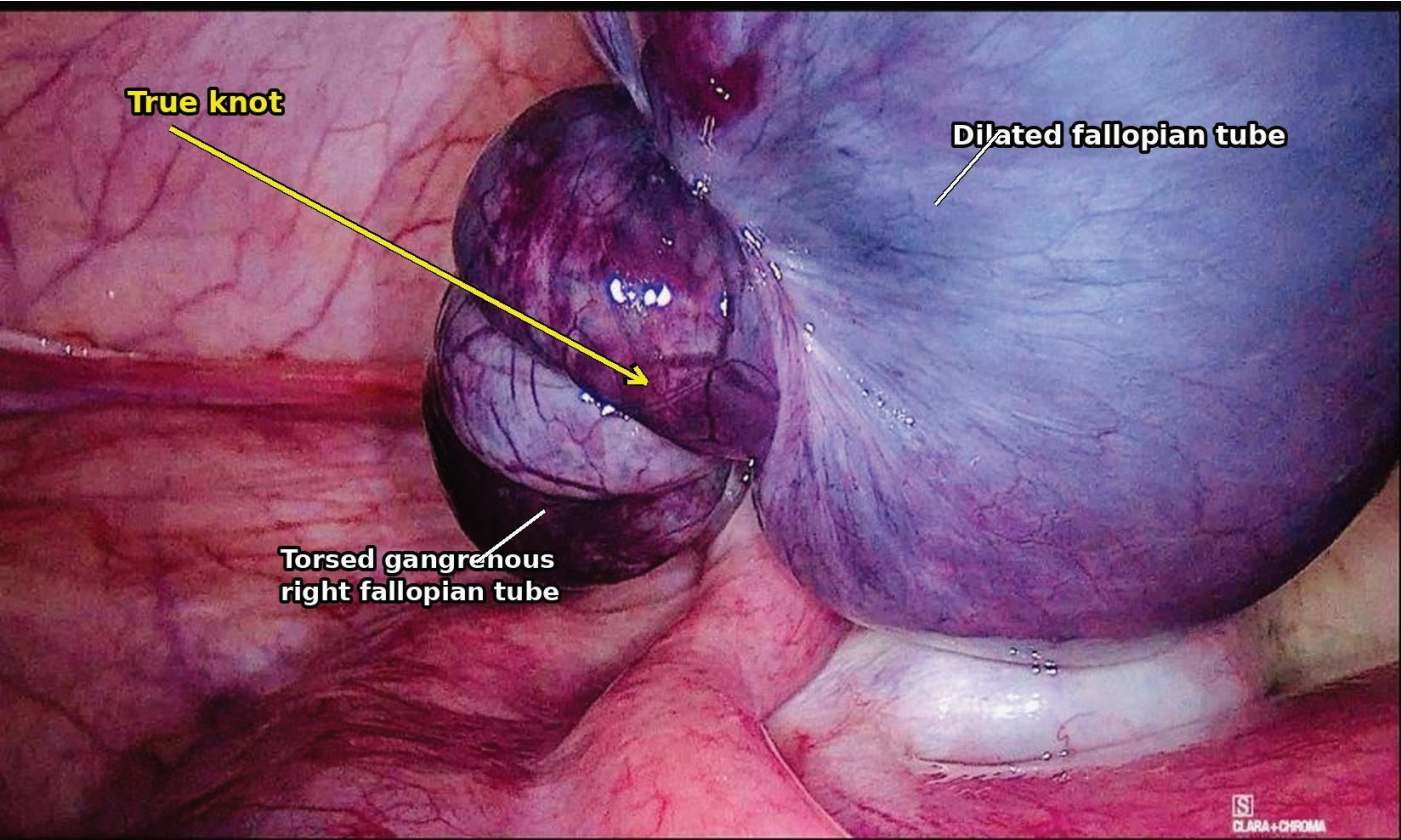
Laparoscopic view demonstrating the true knot formation and advanced gangrene of the right fallopian tube. The yellow arrow indicates the true knot, at the point where the two limbs of the tube pass through the loop; the distended, congested tube and the torsed gangrenous segment are labelled.

**Figure 6:**
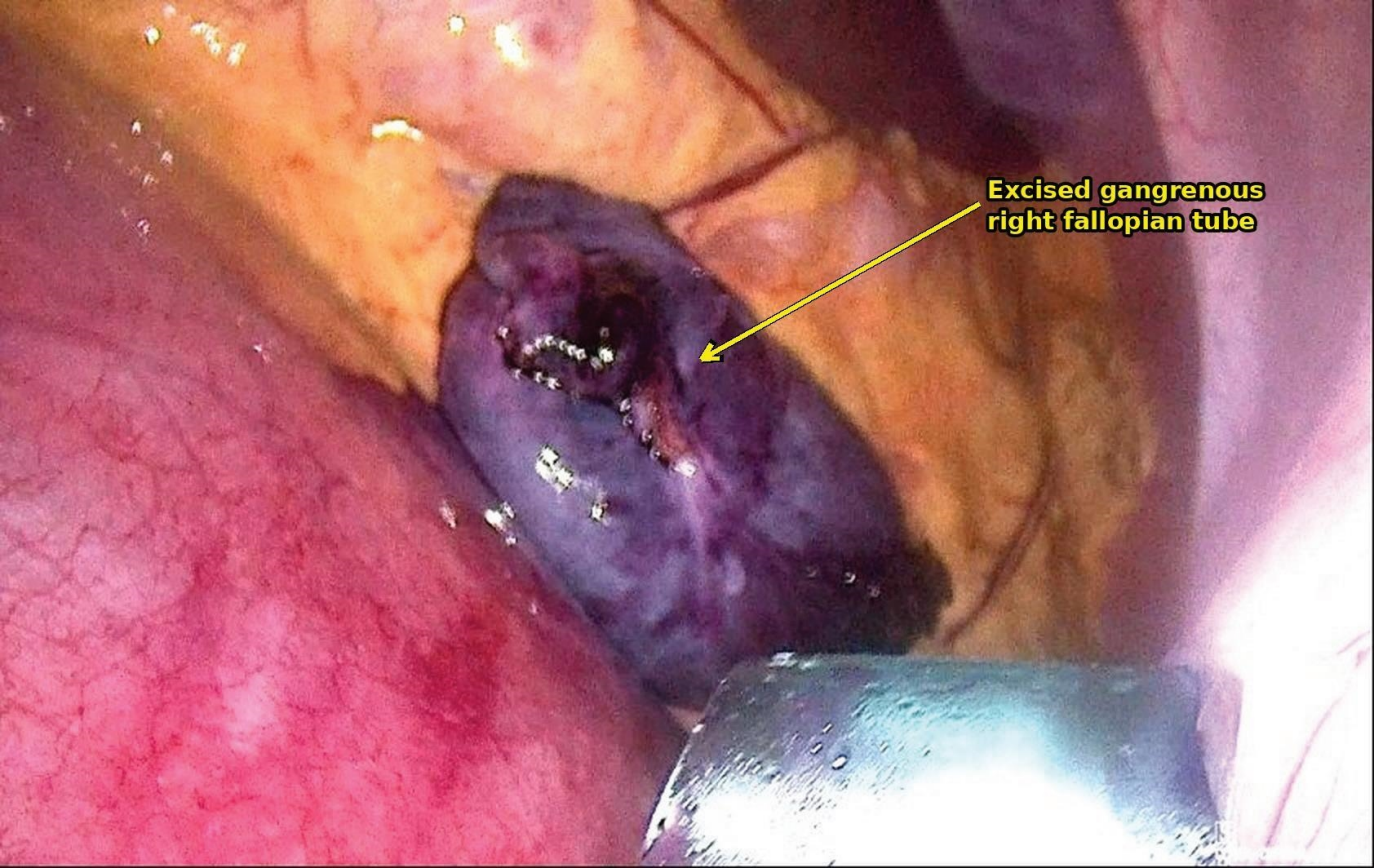
Laparoscopic view of the excised, separated gangrenous right fallopian tube prior to extraction in the Endo Bag. The yellow arrow indicates the excised specimen.

• Operative findings and technique: The patient was placed in the Trendelenburg position under general anaesthesia. Pneumoperitoneum was established using a Veress needle and maintained at a pressure of 12 mmHg. The first 10 mm trocar was inserted at Palmer′s point (left subcostal region) to safely navigate the gravid uterus. A second 10 mm trocar was placed at the umbilicus under direct vision, and a third 5 mm trocar was positioned in the left lateral region. On panoramic laparoscopic inspection, the right fallopian tube was found to be gangrenous and twisted upon itself at least six times with a true knot formation, completely necrotic with no viable tissue (Figures 4, 5 and 6). The right ovary, left adnexa and appendix were all grossly normal, and the pelvis was inspected specifically for predisposing factors such as a paraovarian cyst or adhesions, none of which were present. The right fallopian tube was coagulated below the site of torsion using bipolar diathermy and excised. The specimen was placed in an endo bag and extracted via the umbilical port. Total operative time was approximately 45 min. The uterus was assessed sonographically intraoperatively, confirming a viable fetus. The excised tube was sent for histopathological examination. An intraoperative video recording of the torsed and knotted right fallopian tube is provided as Video S1, and the full‐length recording of the operative findings is provided as Video S2.

Postoperatively, the patient was monitored in the labour room for 6 h. She received an atosiban infusion protocol for 24 h to reduce uterine contractility, along with dexamethasone, ceftriaxone and regular analgesia. Fetal heart rate monitoring confirmed fetal wellbeing throughout. She was clinically stable with a soft abdomen and positive bowel sounds by the evening of surgery. She was discharged home on Day 2 postsurgery on vaginal progesterone pessaries 400 mg twice daily, iron supplementation, calcium and low‐molecular‐weight heparin (enoxaparin 40 mg [0.4 mL] subcutaneously once daily). Outpatient follow‐up was arranged.

**Figure 7:**
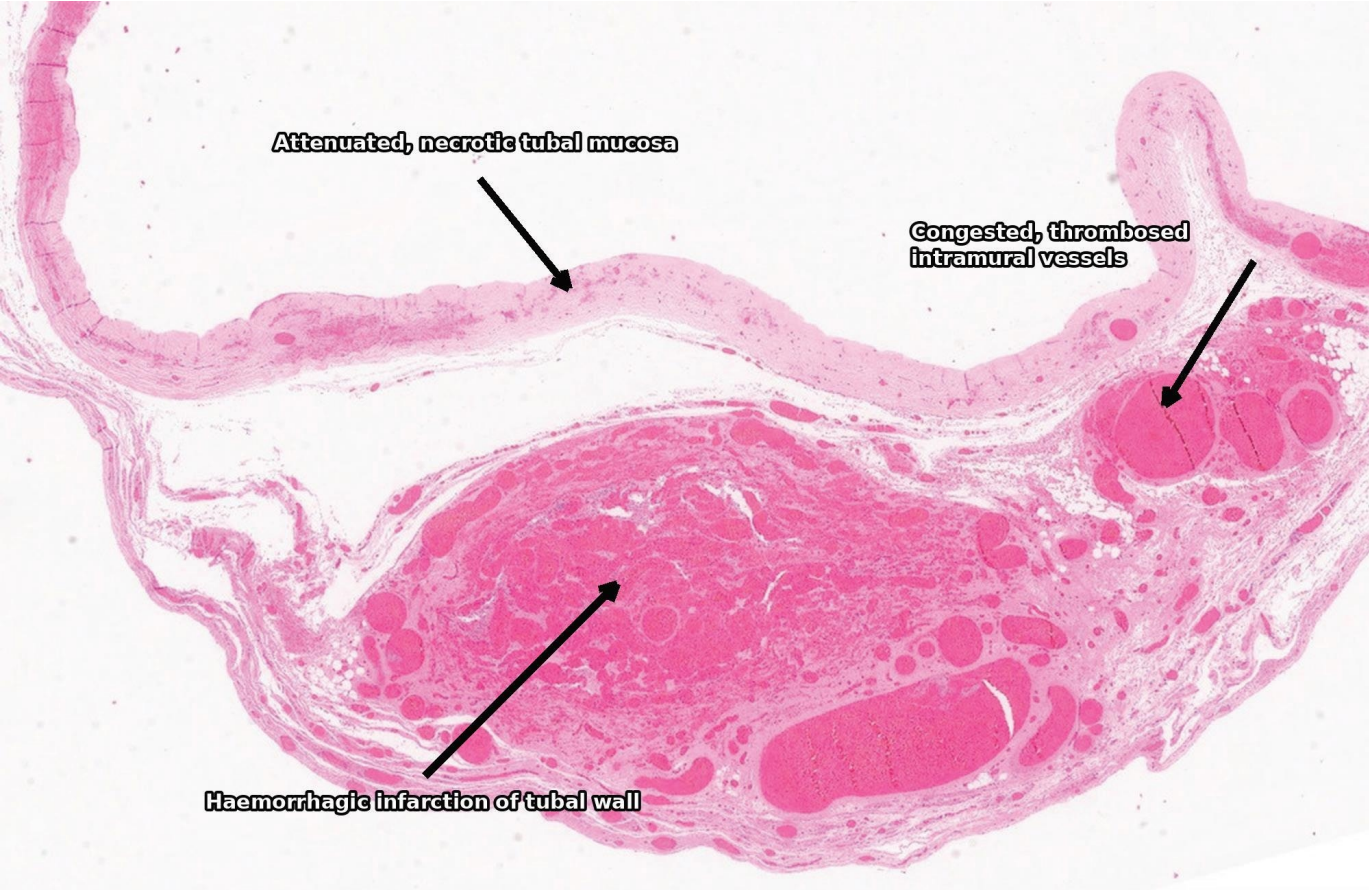
Haematoxylin and eosin–stained section of the excised right fallopian tube demonstrating haemorrhagic infarction with obliteration of normal tubal architecture, congested and thrombosed intramural vessels and attenuated necrotic tubal mucosa, consistent with torsion‐induced ischaemia. Annotations mark each of these three features. Low‐power view; the archived digital image carries no embedded calibration data, and a measured scale bar could therefore not be added. A calibrated image can be supplied by our pathology department on request.

Histopathological analysis of the excised right fallopian tube confirmed haemorrhagic infarction with epithelial necrosis of the tubal mucosa, consistent with torsion‐induced ischaemia (Figure 7). The patient′s antenatal follow‐up was unremarkable. At 40+3 weeks of gestation, she underwent induction of labour and delivered a healthy female neonate weighing 3.385 kg with Apgar scores of 8/9. Postnatal recovery was uneventful.

## 3. Discussion

Isolated fallopian tube torsion is rare, and the pregnancy‐specific literature is smaller than is often assumed. The only population‐based incidence estimate remains Hansen′s Danish survey, which suggested approximately one case per 1.5 million women annually [2]. In pregnancy, fewer than 50 cases have been published [3]: 19 were collected in a review covering 1936 to 2009 [4], and a systematic review of the subsequent decade identified 10 more [5]. A 2024 systematic review of case reports assembled 131 individual cases of IFTT [14]; that review, however, explicitly excluded pregnant patients of all trimesters, so its findings describe the non‐pregnant population and cannot be used to characterise IFTT in pregnancy. This distinction matters when placing the present case in context, and it is the reason we frame our comparison against the pregnancy‐specific literature [3–5, 15].

Torsion predominates on the right, as in our patient, reported in more than 90% of cases in one review and attributed to the sigmoid colon limiting mobility of the left adnexa, whereas the right adnexa lies alongside the more mobile caecum and ileum [3]; an adolescent series has nonetheless reported an equal distribution between sides, so the asymmetry should not be treated as absolute [8]. No predisposing factor was identified in our patient: there was no paraovarian cyst, hydrosalpinx or adhesion. The gravid uterus is itself recognised as a mechanical risk factor, and increased tubal vascularity with elongation of the mesosalpinx in pregnancy may contribute [6, 7]. The intraoperative finding of a true knot is unusual; a knotted configuration is not described as a characteristic operative finding in the pregnancy‐specific reviews [3–5], and we have therefore annotated the operative stills (Figures 4, 5 and 6) and provided the operative video so that the configuration can be assessed directly by the reader.

Clinically, our patient presented with persistent right iliac fossa pain radiating to the right renal angle, refractory to analgesia. This pattern is characteristic but non‐specific, and in this case, was initially attributed to recurrent UTI and pyelonephritis. The same trap has been described previously, in a pregnant patient whose tubal torsion presented as hydronephrosis of pregnancy [16], and the difficulty is general rather than particular to our institution: in a 12‐year series of 17 surgically confirmed cases of IFTT, not one was diagnosed preoperatively [11]. In our patient, approximately 2 weeks elapsed between the onset of pain and laparoscopy. For adnexal torsion generally, a delay of more than 10 h from the onset of pain to surgery has been associated with an increased risk of adnexal necrosis [17], and the complete gangrene found at operation is consistent with prolonged ischaemia.

With respect to imaging, ultrasound with colour Doppler identified a right adnexal cystic lesion in our case, but ovarian vascularity was preserved—a recognised pitfall, because the dual blood supply of the fallopian tube from both the ovarian and uterine arteries means that preserved Doppler flow does not exclude torsion [10]. The whirlpool sign on colour Doppler, representing the twisted vascular pedicle seen between a morphologically normal ovary and the uterus, should raise clinical suspicion [10]. We should be transparent about a limitation of our imaging documentation: The archived static ultrasound image available for publication (Figure 1) records colour Doppler interrogation of the right adnexal region but does not itself resolve the cystic lesion described in the contemporaneous radiology report, and we have written the legend accordingly rather than annotating the image with a finding it does not demonstrate. MRI (Figures 2 and 3) demonstrated the encysted right iliac fossa collection and reported a collapsed appendix. We wish to clarify this term, which is open to more than one reading: It denotes a normal, nondistended appendix, not an appendix that has decompressed following perforation. No MRI feature of appendicitis or of perforation was present, and a macroscopically normal appendix was confirmed at laparoscopy. Although MRI is increasingly valuable in the pregnant patient for excluding appendicitis and characterising adnexal pathology, it did not conclusively diagnose IFTT. Current evidence suggests that the primary value of imaging in this setting lies in systematically excluding other causes of acute pain rather than confirming tubal torsion, which remains a surgical diagnosis [10, 12].

Regarding surgical management, laparoscopy during the second trimester of pregnancy is supported by multiple studies and societal guidelines. The Society of American Gastrointestinal and Endoscopic Surgeons (SAGES) guidelines affirm that laparoscopy is safe across all trimesters when clinically indicated [13]. In our case, we used a pneumoperitoneum pressure of 12 mmHg, which lies within the range considered acceptable in pregnancy, sufficient for adequate visualisation while limiting the risk of fetal compromise from maternal hypercapnia [13]. Palmer′s point entry was used to negotiate the enlarged uterus safely and to avoid uterine injury [13]. Meticulous inspection of the entire pelvis was performed to search for predisposing factors such as paraovarian cysts and pelvic adhesions, none of which were identified. Fetal heart rate was monitored both intraoperatively by ultrasound and postoperatively.

In cases where the tube is viable, detorsion with or without cyst excision may be considered to preserve fertility. In our patient, however, the tube was irreversibly gangrenous with a true knot, and salpingectomy was the only appropriate surgical option. Data on fertility specifically after salpingectomy for tubal torsion are lacking. The closest available evidence comes from the management of tubal ectopic pregnancy, where a systematic review and meta‐analysis found comparable subsequent fertility after salpingectomy and salpingotomy in women with a healthy contralateral tube [18]. Counselling about the potential implications for fertility and the small risk of torsion in the contralateral tube nevertheless remains important in women of reproductive age.

Set against a pregnancy literature of fewer than 50 published cases, the contribution of this report is threefold. First, it documents a true knot with complete gangrene, an operative configuration not described as characteristic in the available pregnancy series, and does so with annotated operative imaging and video rather than by assertion. Second, it illustrates in detail the diagnostic trap of attributing right‐sided pain to urinary infection in pregnancy, with an interval of approximately 2 weeks from symptom onset to surgery and complete tubal necrosis as the consequence. Third, it adds a confirmed obstetric outcome to the small pregnancy series: spontaneous vaginal delivery of a healthy neonate at 40+3 weeks following second‐trimester laparoscopic salpingectomy. Clinicians should maintain a high index of suspicion for IFTT in any pregnant patient with right‐sided pelvic or iliac fossa pain that is refractory to analgesia and accompanied by a non‐diagnostic urine culture.

This report has limitations. It describes a single patient, and no causal inference can be drawn. A gross photograph of the resected specimen was not obtained at the time of surgery; the intraoperative findings are therefore documented by annotated laparoscopic stills, the operative video and histopathology. The archived ultrasound image does not demonstrate the adnexal lesion described in the radiology report, as noted above. The interval to diagnosis reflects the clinical course at a single institution and should not be read as a general estimate of diagnostic delay in IFTT.

## Funding

No funding was received for this manuscript.

## Disclosure

The author takes full responsibility for the content of the manuscript.

## Consent

Informed written consent was obtained from the patient for the surgical procedure and for publication of this case report, including use of clinical images and operative footage.

## Conflicts of Interest

The author declares no conflicts of interest.

## Supporting information


**Supporting Information** Additional supporting information can be found online in the Supporting Information section. Video S1: Intraoperative laparoscopic video demonstrating the torsed right fallopian tube with the true knot. The clip runs for 79 s and shows, in sequence, panoramic inspection of the pelvis, the gangrenous right tube with the knotted configuration and the relationship of the tube to the gravid uterus and the normal right ovary. The final segment of the previously submitted video, which showed out‐of‐focus peritoneal surfaces after the diagnostic findings and added no diagnostic information, has been removed. Video S2: Full‐length intraoperative laparoscopic recording of the operative findings (9 min 17 s; compressed for upload), provided so that the torsed, gangrenous right fallopian tube with the true knot and its relationship to the gravid uterus and normal adnexal structures can be examined in their entirety and without editing.

## Data Availability

The data that support the findings of this study are available in the Supporting Information of this article.
