# Supplementary material for: Pregnancy‐Associated Isolated Fallopian Tube Torsion With a True Knot Managed by Laparoscopic Salpingectomy: A Case Report
Source: Case Rep Obstet Gynecol. 2026 Sep 11;2026:8852614. doi: 10.1155/crog/8852614 (PMC13564104; doi:10.1155/crog/8852614)
Supplement: Supplementary file 1 [file CROG-2026-8852614-s001.zip › Supplementary_Raw_Data_Tubal_Torsion 1.docx]

# Supplementary Data – Raw Clinical Dataset

## Patient Information

| Age | 25 years |
| --- | --- |
| Gravida/Parity | G1P0 (primigravida) |
| Gestational Age at Presentation | 27+6 weeks |

## Presenting Complaint

| Main Symptom | Severe right iliac fossa pain radiating to groin |
| --- | --- |
| Associated Symptoms | Nausea, dysuria |
| Relevant Past History | Recurrent urinary tract infections during pregnancy |

## Clinical Findings

| Vitals | Stable |
| --- | --- |
| Abdominal Exam | Localized right iliac fossa tenderness |
| Other | No guarding or rebound tenderness |

## Laboratory Results

| Hemoglobin | 8.7–10 g/dL |
| --- | --- |
| WBC | 15–16.4 × 10⁹/L |
| Urine Culture | Negative |

Amylase	76 U\L

Lipase	44 U\L

## Imaging

| Ultrasound | Right adnexal cyst 2.5–3 cm with preserved vascularity |
| --- | --- |
| MRI | Right iliac fossa encysted fluid collection; appendix normal |

## Operative Details

| Approach | Laparoscopy |
| --- | --- |
| Ports | Palmer’s point (1st), umbilicus (2nd), left lateral (3rd) |
| Findings | Right fallopian tube torsed, necrotic, gangrenous; ovaries and appendix normal |
| Procedure | Laparoscopic right salpingectomy; specimen retrieved in endobag |

## Outcome

| Parameter | Details |
| --- | --- |
| Immediate | Uneventful recovery |
| Pregnancy Course | Continued to 40+3 weeks |
| Delivery | Spontaneous vaginal delivery of healthy neonate |

### Histopathology Report

**Specimen:** Right fallopian tube, salpingectomy
**Gross Description:** Congested, necrotic right fallopian tube measuring approximately 6–7 cm in length, with haemorrhagic serosa.
**Microscopic Findings:** Sections show fallopian tube with marked haemorrhagic necrosis, dilated congested vessels, and inflammatory infiltrate. No evidence of malignancy.
**Final Diagnosis:** Haemorrhagic infarction and necrosis of right fallopian tube consistent with torsion.
