# Supplementary material for: Pregnancy‐Associated Isolated Fallopian Tube Torsion With a True Knot Managed by Laparoscopic Salpingectomy: A Case Report
Source: Case Rep Obstet Gynecol. 2026 Sep 11;2026:8852614. doi: 10.1155/crog/8852614 (PMC13564104; doi:10.1155/crog/8852614)
Supplement: Supplementary file 1 [file CROG-2026-8852614-s001.zip › aya hassan tube torsion data.pdf]

All Cases View (Doctor's Details)

Civil ID: 299102803081 File No:F1048838 Gender: أنثى

Patient Name: ايه حسن ابراهيم صبره Nationality: مصرى

AYA HASSAN IBRAHIM SABRA

History

Doctor Note

Visit No. 21618554 Visit Type: Casualty Location: Obs & Gyne Emergency NF2

Date: 12-02-2025 06:15 PM User: Houssam Zalzale - Registrar (Obs. & Gyn. - و الولادة النساء)

Visit No. 21679142 Visit Type: Out-Patient Location: Obs. & Gyn

** Doctor Note ** PAT C/O OF LAP
O/E ABD SOFT & LAX
H/O UTI
VS STABLE
FHS POST
PLAN
URINE R& C /S
CYSTONE TAB 2*2
FOLLOW UP

Date: 04-03-2025 11:35 AM User: Ahmed Mohammed - (Obs. & Gyn. - و الولادة النساء)

Visit No. 21679765 Visit Type: Casualty Location: Obs & Gyne Emergency NF2

** Doctor Note ** 25 YEARS FEM, PT, PG 30 +3 WEEKS
POST M LAPAROSCOPIC RT, SALPINJECTOMY
C/O PAIN
MEDICAL-FREE
SURGICAL-PILES, TYMPANOPLASTY
ANC UNIC D
BP-114/72, P 98 , TEMP 36.6
FH + VE
REFERED FROM OPD FOR BUSCOPAN
PT STILL C/O M PAIN AFTER BUSCOPAN
FOR

CBC
LIV, KID URIN R&M C&S
NS
+ PERFALGAN

Date: 04-03-2025 02:17 PM User: DANAH SALEH HAMAD ALHAQQAN - Assistant Registrar ()

Progress Notes

Visit No. 21577862 Visit Type: In-Patient Location: Gyne & Post Natal High Risk C5 NF2

P0+0
LMP 3/8/2024
GA 26+1
ADMITTED AS CASE OF ACUTE PYELONEPHRITIS

C/O RT LOIN PAIN FOR 1 DAY , DYSURIA ASSOCIATED WITH NAUSA , NO VOMITING OR FEVER
IN THE LAST 3 MONTHS SHE HAD H/O RECURRENT UTI , SHE RECIEVED ORAL ZINNAT AND UVAMIN
LAST URINE CULTURE RESULT DONE IN PRIVATE IN JAN: NO GROWTH

PMH: FREE
PSH: PILES , TYMPANOPLASTY
ALLERGIES: NIL

ANC: POLYCLINIC+ PRIVATE
OGTT NOT DONE YET

O/E BP 100/66 PR 83 TEMP 36.7
P/A SOFT LAX ABDOMEN , NO FLANK TENDERNESS , APPRECIATED THE FM , FH+VE 132
NO LKEAKING OR BLEEDING

HB 9.9 WBCS 15.4 URINE R 500 LEU

PLAN:
RI - CROSS MATCH
URINE C/S
BUSCPAN IN IV FLUID TDS FOR 24HRS

Scanned with CamScanner

All Cases View (Doctor's Details)

Civil ID: 299102803081 **File No:**F1048838 **Gender:**أنثى

Patient Name: ايه حسن ابراهيم صبره **Nationality:**مصرى

AYA HASSAN IBRAHIM SABRA

History

Progress Notes

Visit No. 21577862 **Visit Type:** In-Patient **Location:** Gyne & Post Natal High Risk C5 NF2

ROCEPHIN 2 GM OD TILL URINE CULTURE RESULT IS READY
WARD USS IN THE MORNING
CYSTONE TABS 2*2*7
PRIMPRAN INJ SOS
FEFOL CAPS PO OD

Date:02-02-2025 04:11 AM **User:** SAMAR MOHAMED EL HASSAN ALI - Registrar (Obs. & Gyn. - و الولادة النساء)

MR WITH CONSULTANTS AND SR
PT IS VITALLY STABLE
P 83
BP 100/66
T 36.7
FHS +VE
ALT 52
AST 40
HB 9.4
WBC 15
PLAN
OBSTETRIC MEDICINE
US ABDOMEN
CLEXANE 0.4
IV FLUID NS /8HRS
ROCEPHIN 2GM OD IV
FEFOL OD
CA OD
PERFELGAN SOS
KUO

Date:02-02-2025 08:12 AM **User:** FATMA MOHAMMED IBRAHIM AHMED - Registrar ()

obstetric medicine note

dear collague
thank you for your referral

Mrs Aya 25yr lady G1p0+0 prev healthy admitted with acute pyelonephritis
LMP: 3-8-2024 GA26+1

Mrs Aya reports hx of frequent UTI during her marriage, and in this preganancy she had 2 UTI, at 3 months and 2 weeks ago
the 1st UTI urine c/s showed E.coli treated for 5 days with zinnat
2nd ?UTI urine c/s was clean treated with Nitrofirenton for 5 days
today she presented with sudden onset of rt flank pain radiating to the groin, ass with neasue but no vomiting and chills
she thus seeked medical advice immediatly

she denies hx of rash or pruritis

patient reports hx suggestive of hyperemesis gravidarum at the begining of her preganancy associated with high LFTs

PSHx: hemmoroidectomy
allergies: none
home meds: prenatal vitamens

her vitals T 36.7 hr 83 bp 100/66
o/e: looks in pain
chets: clear
abd: s l
skin: no rash

labs
wbc 15 nut 12.2 hb 9.4 plt 258
RFT: unremkable
BP: mg 0.68
LFT: ALT 52 AST 40 ALK 102 GGT 32 tbili3 dbili 0.5
patient scheduled for US abdomen pelvice 5/2/2025

patient started on Rocephin D1

Scanned with CamScanner

All Cases View (Doctor's Details)

Civil ID: 299102803081 File No:F1048838 Gender:أنثى

Patient Name: ايه حسن ابراهيم صبره Nationality:مصرى

AYA HASSAN IBRAHIM SABRA

History

Progress Notes

Visit No. 21577862 Visit Type: In-Patient Location: Gyne & Post Natal High Risk C5 NF2

impression: UTI

Plan with Dr Mrariam :
1. trace CRP PCT and urine c/s
2. continue Rocephin until urine c/s is released for at least 5 days
3. wednesday CBC LFT RFT
4. correct Mg 2 gram mgsulphate in 200ml NS over 2hrs
5. anemia workup : blood film retic b12 folate ferritin
6. OGTT once out of infection
7. send for hpatitis C
8. consider prophylactic abx if confirmed 2 or more attacks of UTI

Date:02-02-2025 11:01 AM User: AMAL KHALED ABDULLAH ALHERRAN - Registrar ()

morning rounds
with dr nabeel
ga 26wks+2
patient stable
fhs+ve
for obstetric u/s today
follow obstetric medicine plan

Date:03-02-2025 07:53 AM User: Saad Abdullah Hanna Yousef - Assistant Registrar (Obs. & Gyn. - النساء و الولادة)

OBSTETRIC MEDICINE FOLLOW UP NOTE

A 25 YEAR OLD PREVIOUSLY HEALTHY, PG AT 26+2 WEEKS GA (LMP: 3-8-2024)

THE PATIENT REPORTED 1 ATTACK OF UTI SINCE HER MARRIAGE, AND ANOTHER 1-2 ? ATTACKS SINCE THE START OF HER PREGNANCY THAT WAS TREATED WITH A COARSE OF ZINNAT AND NITRO.

SHE WAS ADMITTED FOR 1 DAY H/O: RT FLANK PAIN RADIATING TO THE GROIN AND DYSURIA + FOUND TO HAVE MILDLY IMPAIRED TRANSAMINASES

HER ANTENATAL FOLLOW UPS ARE IN PRIVATE CLINIC

TODAY, NO NEW COPMLAINS
VITALLY STABLE

LABS:
URINE C/S: MIXED GROWTH
PCT 0.072
CRP 10

IMPRESSION: UTI + MILDLY IMPAIRED TARNSAMINASES

PLAN:
1. REPEAT URINE C/S WITH PROPPER COLLECTION
2. CONTINUE ROCIPHIN 2 G I.V OD FOR AT LEAST 5 DAYS
3. U/S ABDOMEN (KIDNEY AND LIVER) ON WEDNESDAY
4. CBC/ RFT/ FULL LFT/ PCT ON WEDESDAY
5. OGTT AS OUT PATIENT ONCE OUT OF INFECTION (TO BE FOLLOWED BY OBGYN)
6. TRACE ANTENATAL HEPATITIS WORK UP
7. DETAILED FETAL U/S IF NOT DONE
8. CONCIDER PROPHYLACTIC ABx IF CONFIRMED > ATTACKS OF UTIs
9. WE WILL SEE THE PATIENT ON WEDNESDAY

Date:03-02-2025 10:45 AM User: SARAH H S ALHUWAIS - Specialist (Obstetric Medicine - باطنية الحوامل)

CALLED TO SEE PTN CO RIF PAIN
PA SOFT LAX
MILD RIF TENDERNESS
FOR
PERFLGAN
SURGICAL CONSULTATIOIN TO RO APPENDICITIS

Date:04-02-2025 04:59 AM User: amr Elsayed Ali Eldesouki - Registrar (Gyncology - النساء)

MR WITH CONSULTANTS AND SR
PG, 26+3WKS
PT IS VITALLY STABLE
P 80

Scanned with CamScanner

All Cases View (Doctor's Details)

Civil ID: 299102803081 | File No:F1048838 | Gender: أنثى
Patient Name: ايه حسن ابراهيم صبره | Nationality: مصرى
AYA HASSAN IBRAHIM SABRA

History

Progress Notes

Visit No. 21577862 | Visit Type: In-Patient | Location: Gyne & Post Natal High Risk C5 NF2

BP 100/64
T 36.7
FHS +VE
PT C/O OF RIF PAIN
SUGEON CAME AND ADVISED FOR URGENT US TO EXCLUDE APPENDICITIS
CONTINUE SAME PLAN

Date: 04-02-2025 07:49 AM | User: FATMA MOHAMMED IBRAHIM AHMED - Registrar ()

RADIOLOGIST ON CALL CONTACTED AND AGREED TO DO THE US

Date: 04-02-2025 07:54 AM | User: FATMA MOHAMMED IBRAHIM AHMED - Registrar ()

SEEN BY SURGICAL ON CALL
:PT ADMITTED 2 YS AGO AS CASE OF LOIN PAIN AND LOWER ABDOMINAL PAIN
PATIENT GIVING LONG HISTORY SINCE MARRIAGE OF RECURRENT UTI
NOW 2 DS PAIN INCREASED MAINLY LOIN PAIN RAD DOWN TO VAGINA ASS WITH N V AND DYSUREA AND DIFFICULT MICTURATION
PAIN ON AND OFF NOT CONTINUOSE RELIEF BY PAIN KILLER NOT ASS WITH ANOREXIA

VITALLY STABLE AFEBRILE :MR WITH CONSULTANTS
P 80
BP 100/64
T 36.7

ABD TENDER LOIN DOWN TO RT ILIAC AND SUPRAPUBIC

LABS :UTI :LECOCYTOSIS IN URINE
WBC 12 WITH NEUTROPHILIA
CRP 10

PROVISIONAL :UTI ?BACK PRESSURE
FOR URGENT US
IF US UNREMARKABLE WILL NEED MRI

KEEP PT FASTING ON IVF 120 ML PER H RL WITH PAIN KILLER

SEND FOR SURGERY IF REASSESSMENT NEEDED

Date: 04-02-2025 11:23 AM | User: WALID BADR IBRAHIM ABUSHANAB - Senior General Practitioner B ()

RADIOLOGY DR CONTACTED DR WALAA
AND AGREE FOR TRANSFER PT FOR MRI
PT REFUSE TO DO MRI
ALL RISK EXPLAINED TO PT AND SHE DIDNOT WANT TO DO MRI UPON HER RESPONSIBILITY

Date: 04-02-2025 01:01 PM | User: BASEM ALI SHEHATA ZAITOUN - Registrar ()

both pt and her husband signed refusal form
dr walwwd surgeun contacted and he said no intervention will be done until mri is done
and pt insisting on refusal

Date: 04-02-2025 01:54 PM | User: FATMA MOHAMMED IBRAHIM AHMED - Registrar ()

SURGICAL UPDATES :
US UNREMARKABLE CONCERNING APPENDIX AND KIDNEY URETER
PT REFUSED MRI TO DO

SO :
CONTINUE ANTIBIOTIC TTT
FOLLOW UP CBC ,CRP

Date: 04-02-2025 02:04 PM | User: WALID BADR IBRAHIM ABUSHANAB - Senior General Practitioner B ()

ROUND WITH DR NABIL . DR GHARESA . DR FADL , DR SANAA . DR MAHMOUD
today pt is doing well
VIT STABLE. AFEBRILE
FHS + VE
ABD SOFT
NO LAP , NO VAG BLEEDING
URINE CULTURE CAME . NO GROWTH
HB 9.7 GM WBC 12.2, CRP 6
PT IS FOR DISHARGE FROM OUR SIDE
TO BE REVIEWED BY OBST MED TEAM BEFORE DISHARGE

Scanned with CamScanner

All Cases View (Doctor's Details)

Civil ID: 299102803081 File No:F1048838 Gender:أنثى

Patient Name: ايه حسن ابراهيم صبره Nationality:مصرى

AYA HASSAN IBRAHIM SABRA

History

Progress Notes

Visit No. 21577862 Visit Type: In-Patient Location: Gyne & Post Natal High Risk C5 NF2

OPD NEXT WEEK WITH UNIT D
SICKE LEAVE FOR ONE WEEK
DISHARGE ON :
ZINNAT TAB 500 MG TDS /7 D
FEFOL TAB DAILY
CAL TAB DAILY
BUSCUBAN TAB BD /5 D

Date:05-02-2025 07:55 AM User: Wael El-Shabrawy - Registrar (Obs. & Gyn. - و الولادة النساء)

obstetric medicine follow up note

dear collague
thank you for your referral

Mrs Aya 25yr lady G1p0 GA26+4 prev healthy admitted with UTI
LMP: 3-8-2024

Mrs Aya reports hx of frequent UTI during her marriage, and in this preganancy she had 2 UTI, at 3 months and 2 weeks ago
the 1st UTI urine c/s showed E.coli treated for 5 days with zinnat
2nd ?UTI urine c/s was clean treated with Nitrofirenton for 5 days
today she presented with sudden onset of rt flank pain radiating to the groin, ass/ with nausea but no vomiting and chills
she thus seeked medical advice immediatly

she denies hx of rash or pruritis

patient reports hx suggestive of hyperemesis gravidarum at the begining of her preganancy associated with high LFTs

PSHx: hemmoroidectomy
allergies: none
home meds: prenatal vitamens

her vitals T 36.7 hr 83 bp 100/66
o/e: looks in pain
chets: clear
abd: soft and lax
skin: no rash

labs
wbc 15 >>12 nut 12.2>9 hb 9.4>> 10 plt 258
RFT: unremkable
BP: mg 0.66
LFT: ALT 52 >>42 AST 40>>36 ALK 102>>106 GGT 32 tbili3 dbili 0.5
PCT 0.091 CRP 6
ferritin 6.1
urine c/s no growth

US abdomen was unremkable

patient started on Rocephin D4

impression: UTI with anemia

Plan with Dr Sheikha:
1. discharge patient with follow up urine c/s after 1 week of completion of antibiotics course, to follow up result with OBGYN OPD.
2. discharge patient on oral iron supplements (globifer) repeat Ferritin after 2 months, if still low to give IV iron.
3. kindly give 2gram mgsulphate in 200ml ns over 2 hours STAT before discharge
4. case signed off.

Date:05-02-2025 09:54 AM User: AMAL KHALED ABDULLAH ALHERRAN - Registrar ()

addendum to obstetric medicine plan
patient adviced to repeat OGTT in polyclinc after discharge

Date:05-02-2025 10:03 AM User: AMAL KHALED ABDULLAH ALHERRAN - Registrar ()

surgical team unit c dr walid senior and dr mhmoud specialist came to assess patient we found patient already discahrged from ward by gyne team

Date: User: WALID BADR IBRAHIM ABUSHANAB - Senior General Practitioner B ()

Scanned with CamScanner

All Cases View (Doctor's Details)

Civil ID: 299102803081 File No:F1048838 Gender: أنثى

Patient Name: ايه حسن ابراهيم صبره Nationality: مصرى

AYA HASSAN IBRAHIM SABRA

History

Progress Notes

Visit No. 21577862 Visit Type: In-Patient Location: Gyne & Post Natal High Risk C5 NF2

05-02-2025 11:00 AM

Visit No. 21619109 Visit Type: In-Patient Location: Gyne & Post Natal High Risk C5 NF2

25yr lady G1p0+0 prev healthy admitted AS K.C.O UTI WITH RT LOIN PAIN NOT RESPONDING TO ORDINARY ANALGESIA
LMP: 3-8-2024 GA27W+4

PT. GAVE H.O of frequent UTI during her marriage, and in this preganancy she had 2 UTI, at 3 months and 2 weeks ago
SHE WAS ADMITTED ON 2/2/2025 AS A CASE OF AC. PYLONEPHRITIS SEEN BY OBST MEDICINE & LABELLED AS UTI WITH ANAEMIA
U/S DONE : ??The liver is of average size, it displays homogenous parenchymal echo-pattern. No focal lesions or intra-hepatic biliary tree dilatation.
Normal caliber of the portal vein.
??Average sized gall bladder. No echogenic stones or mud inside. Normal CBD.
??The spleen is of average size. It is of homogenous echo-pattern and no focal lesion.
??Either kidney is of average size, normal site and contour as well as normal renal parenchymal thickness and echogenicity. No renal stones or back-pressure changes.
??The pancreas is masked by colonic gases.
??No ascites or pelvic collection.
??Empty urinary bladder.
??Gravid uterus with viable fetus inside.
??The appendix can?t be visualized.
??Bulky right ovary with a well-defined right adnexal cystic lesion measures about 2.5 x 2.4 cm.
PT. OFFERED MRI FOR FURTHER EVALUATION BUT SHE REFUSED
- DISCHARGED ON ZINNAT ,+CYSTON WITH F/U IN OPD & TO REPEAT URINE C/S BUT SHE DIDNOT ATTEND
the 1st UTI urine c/s showed E.coli treated for 5 days with zinnat
2nd ?UTI urine c/s was clean treated with Nitrofirenton for 5 days
today she presented with sudden onset of rt flank pain radiating to the groin, ass with neasue & VOMITING vomiting
she RECIEVED BUSCOPAN +IVF ,PERFALGAN IN CASUALTY BUT PAIN NOT SUBSIDED

PMH : -VE
PSHx: hemmoroidectomy ,TYMPANOPLASTY
allergies: none
home meds: prenatal vitamens

her vitals: T 36.9 PULSE : 86 bp 95/70 SAT O2 : 100% (ROA)

ABD. SOFT & LAX ,MILDLY TENDER RT RENAL ANGLE
MILD TENDERNESS ON RIF BUT NO RIGIDITY
GOOD FM FHS : +VE
OBST U/S (3/2/2025) : SVF ,N. LIQ. ,UPPER POST. PLACENTA ,26W+6/26W+2,EFW : 993 GM
LAB : HB : 9.7 WBCS : 16.4
LFT ,RFT STILL PEDING
FOR : KUO
IVF 125ML /H
BUSCOPAN AMP SOS
FOLLOW RESULT OF RFT & LFT
CRP
URGENT TAS (RADIOLOGY DEPARTMENT)
INFORM IF PAIN INCREASING

Date:12-02-2025 11:12 PM User: Amany Elsayed Mosad Elsayed Younis - Registrar (Obs. & Gyn. - النساء و الولادة)

I CAME TO SEE PAT
C/O OF LAP
BP 95/70 P 86 T 36.9
US DONE RT SMALL SIMPLE CYST WITH NO TORSION
URINE R LEU 500
PA RT LOIN PAIN + RT ILIAC FOSSA PAIN

PLAN
START 2 GM ROCEPHIN OD
125 ML IV FLUID/H
CYSTONE 2*2*14
KEEP NPO
URINE CULTURE
BUSCUPAN IM
SURGICAL CONSULTATION

Scanned with CamScanner

All Cases View (Doctor's Details)

Civil ID: 299102803081 File No:F1048838 Gender: أنثى

Patient Name: ايه حسن ابراهيم صبره Nationality: مصرى

AYA HASSAN IBRAHIM SABRA

History

Progress Notes

Visit No. 21619109 Visit Type: In-Patient Location: Gyne & Post Natal High Risk C5 NF2

Date: 13-02-2025 03:51 AM User: Ahmed Mohammed - (Obs. & Gyn. - و الولادة النساء)

s o c unit f:
we were notified to assess the patient;pregnant at27 weeks, with H.O of frequent UTI during her marriage, and in this preganancy she had 2 UTI, at 3 months and 2 weeks ago
SHE WAS ADMITTED ON 2/2/2025 AS A CASE OF AC. PYLONEPHRITIS SEEN BY OBST MEDICINE & LABELLED AS UTI WITH ANAEMIA
ror: rt sided abdominal pain since one day associated with n , v
no fever no dysuria
o/e:
vitally stable , afebrile
abdomen: tender rt side of abdomen with rebound tenderness , no crossed rebound
u/s:
The gall bladder is adequately distended with normal wall thickness and small echogenic lesions seen related to the wall, largest measures 5 mm, impressive of polyps no evidence of calculus or sludge.
No ascites or abdominal collections.
Small right ovarian cyst of simple nature.
Appendix couldn?t be visualized.
labs:
wbc 16.4, hb 9.7
urine: leu 500, nit negative
impression: appendicitis vs uti
recommendations:
-crp, lactic acid
-urgent amylase and lipase
-urgent mri to r/o appendicitis
-urine c/s
-pain control as per treating unit

Date: 13-02-2025 04:38 AM User: AHMED HALIM MOHAMED AHMED ELHINDAWI - Senior General Practitioner B ()

patient is pain
looks distressed
recieved perphelgan at 2 am
and buscopan recieved 30 minutes
initially patient refused mri for financial reasons
plan :
patient is for pethidine 50mg im once pain reliefed patient to be evaluated again and proper councelling to be done

Date: 13-02-2025 05:52 AM User: Saad Abdullah Hanna Yousef - Assistant Registrar (Obs. & Gyn. - و الولادة النساء)

MORNING ROUND

PG , 27 WEEKS ADMITTED WITH RT LOIN PAIN
TODAY PATIENT IS STILL COMPLAINING OF PAIN RECIVED INJ PETHADINE
VITALLY STABLE
ABD SOFT WITH MILD TENDERNESS

PATIENT COUNSELED REGARDING MRI AND AGREED CONSENT SIGNED , RADIOLOGIST ACCEPTED TO DO IT URGENTLY
SURGICAL ON-CALL CONTACTED ADVISED FOR ANESTHESIA AND OT BOOKING FOR POSSIBLE DL

Date: 13-02-2025 09:25 AM User: RAGHAD SAMI BO GHAIS - Registrar (Obs. & Gyn. - و الولادة النساء)

DR FAHMY SURGERY CONTACTED ME REGARDING THE PT AND SAID THAT HE CONSULTED RADIOLIGY CONSULTANTS REGARDING THE MRI IMAGING AND THEY CONFIRMED IT IS NOT APPENDICITIS
DR FAHMY RECOMMENDED TO KEEP THE PT NPO AND GIVE IV ANTIBIOTICS TILL THEY COME ASSESS HER TOMORROW

Date: 13-02-2025 02:01 PM User: FATMA MOHAMMED IBRAHIM AHMED - Registrar ()

PT HAS HX OF RCURRENT UTI

Date: 13-02-2025 02:03 PM User: FATMA MOHAMMED IBRAHIM AHMED - Registrar ()

MRI DONE AND THEY VERBALLY EXCLUDED APPENDISOITIS
KUO , NPO, FOR SURGICAL ASSESSMENT TOMORRROW BAD SURGERY RECOMMENDED

Date: 13-02-2025 02:03 PM User: FATMA MOHAMMED IBRAHIM AHMED - Registrar ()

P1, 27 WKS , RIF PAIN Q APPENDISITIS

Date: 13-02-2025 02:03 PM User: FATMA MOHAMMED IBRAHIM AHMED - Registrar ()

SURGICAL NOTES:

MRI DONE : ??Right iliac fossa encysted fluid collection , likely adnexal in origin

Scanned with CamScanner

All Cases View (Doctor's Details)

Civil ID: 299102803081 File No:F1048838 Gender: أنثى

Patient Name: ايه حسن ابراهيم صبره Nationality: مصرى

AYA HASSAN IBRAHIM SABRA

History

Progress Notes

Visit No. 21619109 Visit Type: In-Patient Location: Gyne & Post Natal High Risk C5 NF2

??Suspected collapsed appendix with no radiological evidence of active inflammatory changes could be appreciated

PLAN WITH DR BADER:
CONSERVATIVE MANAGEMENT
KEEP PT NPO TODAY
IVF 120 ML/HR NS
CONTINUE ROCEPHIN

CBC, CRP TOMORROW 6 AM
WE WILL FOLLOW THE PT

Date: 13-02-2025 02:31 PM User: MOHAMED FAHMY HASSAN ELNEMR - Senior Registrar (General Surgery - الجراحة العامة)

PATIENT SEEN
PATIENT LOOKS WELL AND V/S
ABD SOFT AND LAX
MILD RIF PAIN

PLAN
BY S/R DR BATLA
TO FOLLOW SURGICAL PLAN
INFORM IF ANY EMERGENCY

Date: 13-02-2025 09:07 PM User: AHMAD NABEEL ABDULMOGHNI MOHAMMAD - Registrar ()

MORNING ROUND UNIT A

PRIMIGRAVIDA GA 28WKS 3 DAYS
ADMITTED DUE TO PAIN IN RIGHT ILIAC FOSSA
PT WAS ADMITTED ON 02/02/2025 AS A CASE OF PYELONEPHRITIS
VITALS
BP 101/68 P 72 T 37.0
FHS +VE
MRI RT ILIAC FOSSA ENCYSTED FLUID COLLECTION ?? COLLAPSED APPENDIX
SURGERY ON CALL ADVISED FOR CBC CRP URINE CULTURE IN MORNING
THEN THEY WILL FOLLOW UP PT
PT COUNSELLED TO SEND INVESTIGATION AS SHE IS REFUSING FOR IT
PLAN CONTINUE SAME TREATMENT

Date: 14-02-2025 09:09 AM User: AISHA RAFIQUE - Registrar ()

SURGICAL NOTES WEEKEND NOTES

MRI DONE : ??Right iliac fossa encysted fluid collection , likely adnexal in origin
??Suspected collapsed appendix with no radiological evidence of active inflammatory changes could be appreciated

PT COMOALLING OF PAIN ON AND OFF ,
VITALLY STABLE AFEBRILE
ABD SOFT LAX
MILD RIF TENDERNESS

CBC CRP RESULT PENDING TP BE FOLLOWED TO SEND TO DR MONA 99954245

PLAN WITH DR BADER:
CONSERVATIVE MANAGEMENT
CONTINUE ROCEPHIN
CAN START ORAL FLUIDS

CBC, CRP TOMORROW 6 AM

Date: 14-02-2025 10:35 AM User: MONA ADEL ALI ALMAQSEED - Assistant Registrar ()

I CALLED TO SEE PG 28+3 WKS ADMITTED WITH RIF PAIN C/O PAIN
MRI ?Right iliac fossa encysted fluid collection , likely adnexal in origin
??Suspected collapsed appendix with no radiological evidence of active inflammatory changes could be appreciated

SEEN BY SURGICAL ONCALL TODAY MORNING ADVICED FOR:

CONSERVATIVE MANAGEMENT
CONTINUE ROCEPHIN
CAN START ORAL FLUIDS

Scanned with CamScanner

All Cases View (Doctor's Details)

Civil ID: 299102803081 File No:F1048838 Gender:أنثى
Patient Name: ايه حسن ابراهيم صبره Nationality:مصرى
AYA HASSAN IBRAHIM SABRA

History

Progress Notes

Visit No. 21619109 Visit Type: In-Patient Location: Gyne & Post Natal High Risk C5 NF2

PT RECIVED PERFELGAN IVD WITHOUT IMPROVEMENT
FOR PETHIDINE 50 MG IM STAT ,
PERFELGAN 1 GM / 8 HR IVD
CONTINUE ANRIBITICS
TO BE ASSESSED BY SURGICAL
FOLLOW SURGICAL ORDER

Date: 14-02-2025 01:21 PM User: ASMAA ABDELBADEA MOHAMED SALEM - Registrar (Obs. & Gyn. - و الولادة النساء)

FOR SURGICAL CONSULTATION FOR REEVALUATION AS PT STILL C/O SEVER PAIN , NOT IMPROVED BY ANALGESIC

Date: 14-02-2025 01:37 PM User: ASMAA ABDELBADEA MOHAMED SALEM - Registrar (Obs. & Gyn. - و الولادة النساء)

Aa 25 year old lady
pg
lmp 3/8/2024
27+6wks

admitted with severe RIF pain

pmh -ve
psh piles, tympanoplasty
nka
blood transfusion -ve

patient seen today
c/o severe RIF, recieved perfalgan and pethidine alternatve and frequently, pain not improved
today :
- no nausea or vomiting
- no bowel symptoms
- no pv loss, no labor pain
- good fm
- mild dysuria
last meal was 2 days back

on examination :
drowsy, anxious
vital signs stable
abdomen s/l with +ve RIF tenderness

investigations :
- all recent reviewed (hb 8.7, wbc 12)
- mri done showed:
Conclusion:
??Right iliac fossa encysted fluid collection , likely adnexal in origin , kindly compare with U/S results.
??Suspected collapsed appendix with no radiological evidence of active inflammatory changes could be appreciated , for clinical and laboratory correlation.
- u/s abd/pelvis:
Right ovarian cyst measures about 3 cm. Intact ovarian vascularity, no evidence of torsion.
Left ovary couldn?t be visualized.

plan :
dr.abdultawab informed about case details, he will review the case for final decision
keep 2 prbc ready
keep npo

Date: 14-02-2025 03:08 PM User: Asmaa Ghazi Benawadh - Registrar (Obs. & Gyn. - و الولادة النساء)

PT SEEN BY CONSULTANT DR AHMAD ABDELTAWAB AND SR DR ASMAA
PT FOR DIAGNOSTIC LAPAROSCOPY, ALL RISK EXPLAINED TO PT AND HER HUSBAND
TRANSFUSION 1 PINT PRBCS BEFOR SHIFTING THE PT TO OT
NEONATOLOGY INFORMED BY DR ASMAA SR
NEONATAL CONSULTATION TO BE SEND
NEW CONSENT TAKEN

Date: 14-02-2025 05:49 PM User: ASMAA ABDELBADEA MOHAMED SALEM - Registrar (Obs. & Gyn. - و الولادة النساء)

FOR DEXA PROTOCOL

Date: 14-02-2025 05:56 PM User: ASMAA ABDELBADEA MOHAMED SALEM - Registrar (Obs. & Gyn. - و الولادة النساء)

SURGICAL NOTES:
I JOINED GYNE TEAM DURING DL

Scanned with CamScanner

All Cases View (Doctor's Details)

Civil ID: 299102803081 File No:F1048838 Gender:أنثى

Patient Name: ايه حسن ابراهيم صبره Nationality:مصرى

AYA HASSAN IBRAHIM SABRA

History

Progress Notes

Visit No. 21619109 Visit Type: In-Patient Location: Gyne & Post Natal High Risk C5 NF2

INTRAOP FINDINGS : TORTION GANGRENOUS RT FALOPIAN TUBE : EXCISED BY GYNE TEAM
CEACUM AND APPENDIX APPEAR NORMAL
NO SURGICAL ISSUE FROM OUR SIDE
PLEASE FOLLOW GYNE ORDERS

Date:14-02-2025 08:01 PM User: MOHAMED FAHMY HASSAN ELNEMR - Senior Registrar (General Surgery - الجراحة العامة)

post laparoscopic Rt salpingectomy

seen in LR for observation
Doing well
mild LAP
received Pethidine 100mg
on Tractocele
Received dexamethasone first dose
received 1 pint PRBC

V/S: BP 100/60 HR 95 Afebrile
FHS: 136

plan:
- shift to ANW
- CBC after 6 hrs
- continue course of Dexa
- stop tractocele
- start cycolgest 400mg BD
- continue rocephin 2gm OD for 2 days
- perfelgan 1gm iv tds

Date:14-02-2025 11:48 PM User: HESSAH ABDULRAHEEM NAJEM ALRASHED - Senior Registrar (Obs. & Gyn. - النساء و الولادة)

REASSESSMENT
PT LOOKS FINE
C/O MILD ABDOMENAL PAIN (INSCIONAL SITE)
V/S: BP: 95/60 HR:82 TEMP:36.8C
FHS+VE 148
ABD SOFT AND LAX
+VE BOWEL SOUND
HB:"10

PLAN:
D/C IVF
D/C CATHATER
AMBULATION
START FLUID DIET
PERFELGAN 1 GM SOS
LACTLOSE HS
CONTINIUE SAME PLAN

Date:15-02-2025 09:30 AM User: DANA MOUSA M ALOBAIDAN - Assistant Registrar (Obs. & Gyn. - النساء و الولادة)

VTE SCORE IS 3
START CLEAXE 0.4MG OD

Date:15-02-2025 09:34 AM User: DANA MOUSA M ALOBAIDAN - Assistant Registrar (Obs. & Gyn. - النساء و الولادة)

POST LAPAROSCOPIC SALPENGECTOMY (TORSION OF TUBE)
PT IS WELL
VITALLY STABLE
ABD SOFT & LAX
FHS +VE
NO P/V LOSS
HB (10) BG : O+VE
WOUND DRESSING IS DRY & CLEAN
PT. RECIEVED DEXA & TRACTOCIL
FOR : TAS
DISCHARGE
CYCLOGEST 400 MG SUPP 1*2*14
FEFOL CALCIUM ADOL TAB
CLEXAN 0.4 ML ,OD
OPD F/U AFTER 2W
ATTEND MAT. CASUALTY SOS IF PAIN ,LEAKING OR BLEEDING

Date:

Scanned with CamScanner

All Cases View (Doctor's Details)

Civil ID: 299102803081 | File No:F1048838 | Gender:أنثى

Patient Name: ايه حسن ابراهيم صبره | Nationality:مصرى

AYA HASSAN IBRAHIM SABRA

History

Progress Notes

Visit No. 21619109 | Visit Type: In-Patient | Location: Gyne & Post Natal High Risk C5 NF2

16-02-2025 08:42 AM | User: Amany Elsayed Mosad Elsayed Younis - Registrar (Obs. & Gyn. - و الولادة النساء)

Visit No. 21919780 | Visit Type: In-Patient | Location: Gyne C6 NF2

25 y/o egyptian lady
PG 40w3d admitted on 12/5 for iol today

med hx free
surg hx tympoanoplasty , heamorroidectomy, rt laparoscopic salpingectomy for torted fallopian tube 2 months back
allergic hx free
anc unit D

pt is v.stable
P/A s&I , SFH going with term
fhs +ve today 135 bpm
O+ve , hb 12,.3 gm/dl
screeing -ve
NST just started

plan:
u/s before iol
inform when u/s ready
iol with propess

Date: 13-05-2025 08:18 AM | User: Habayeb abdulamir Matar Marzouq - Registrar (Obs. & Gyn. - و الولادة النساء)

u/s done single living cephalic ,placenta upper ant , oligohydraminos ,GA 38w6d/40w3d
ua ri 0.61

iol with propess doen
p/v cx post 50 % eff soft os 1-2 cm ceph -4 mi , sweeping done
propess applied

Date: 13-05-2025 08:47 AM | User: Habayeb abdulamir Matar Marzouq - Registrar (Obs. & Gyn. - و الولادة النساء)

pt c/o lap ,screaming
pv done cx post 70 %eff soft os 2 cm mi vx -4

plan;
transfer to labour room for pain mangement and prolonged obsersvsation

Date: 13-05-2025 09:43 AM | User: Habayeb abdulamir Matar Marzouq - Registrar (Obs. & Gyn. - و الولادة النساء)

post nvd on 13/5 at 7:01 pm
outcome alive girl wt 3.385 kgs A/S 8/9
pt is well,v.stable
o+ve hb 12.3
normal lochia
P/A s&I ,ut contracted
breast feeding intiated
both LL are normal ,on signs of DVT

plan;
kuo
inform if severe bleeding ,lap ,fever ,abnormal signs and symptoms

Date: 14-05-2025 11:25 AM | User: Habayeb abdulamir Matar Marzouq - Registrar (Obs. & Gyn. - و الولادة النساء)

breast feeding consultation

Date: 14-05-2025 11:38 AM | User: Habayeb abdulamir Matar Marzouq - Registrar (Obs. & Gyn. - و الولادة النساء)

post nvd on 13/5 at 7:01 pm
outcome alive girl wt 3.385 kgs A/S 8/9
pt is well,v.stable
o+ve hb 12.3
normal lochia
P/A s&I ,ut contracted
breast feeding intiated
both LL are normal ,on signs of DVT
vte score 0

Scanned with CamScanner

Ministry of Health
Farwaniya Hospital

Radiologic Consultation Request

Patient Identification :

Patient Name : AYA HASSAN IBRAHIM
English Name : AYA HASSAN IBRAHIM
Nationality : مصرى
Cons. No : 21619109
Visit Type : InPatient
Clinic :

Sex: Female
Cons. Date: 12-02-2025
Ward : Gyne & Post Natal Hig   Room : 3

File No : F1048838
Civil ID : 299102803081
Marital Status : متزوج
Age : 25 Years -- 10 Months
Bed : 2

Helpful Information For X-Ray:

Bronchial asthmal : No   Diabetes : No
Pregnancy : No   S.Creatinine :

Specific Reason (s) for Request :

PG, 27WKS, RT LOIN PAIN

Case View :

PERSISTAN RT LOIN PAIN REFERRED TO RIF & SPRAPUBIC REGION NOT RESPONDING TO SIMPLE ANALGESIA

Examination Required :

Ultrasound Abdomen + Pelvis

*Signature & Stamp

Dr. Amany Elsayed Mosad Elsayed Younis

Report :   Date : 13-02-2025

Ultrasound Abdomen + Pelvis of 13-FEB-2025:
History Provided:
Findings:

??The liver is of normal size and shape with smooth contour and normal homogenous echo texture.
??The course and caliber of the common and intra hepatic biliary ducts are normal
??The gall bladder is adequately distended with normal wall thickness and small echogenic lesions seen related to the wall, largest measures 5 mm, impressive of polyps no evidence of calculus or sludge.
??Normal size \T\ configuration of both kidneys with good cortical thickness without stones or hydronephrosis.
??The spleen has normal sonographic configuration.
??No ascites or abdominal collections.
??The pancreas was masked.
??Normal volume of the urinary bladder with smooth wall thickness.
??Gravid uterus with viable fetus inside.
??Small right ovarian cyst of simple nature.
??Appendix couldn?t be visualized.

Additional Report 13-FEB-2025, 03:29 AM:

Right ovarian cyst measures about 3 cm. Intact ovarian vascularity, no evidence of torsion.
Left ovary couldn?t be visualized.

* This document is digitally signed, No need for Dr. Signature.

* هذه الوثيقة موقعة إليكترونيا ولا تحتاج إلى توقيع من الطبيب.

Scanned with CamScanner

Ministry of Health
Farwaniya Hospital

Radiologic Consultation Request

Patient Identification :

Patient Name : AYA HASSAN IBRAHIM
English Name : AYA HASSAN IBRAHIM
Nationality : مصرى
Cons. No : 21619109
Visit Type : InPatient
Clinic :

Sex: Female
Cons. Date: 12-02-2025
Ward : Gyne & Post Natal Hig   Room : 3

File No : F1048838
Civil ID : 299102803081
Marital Status : متزوج
Age : 25 Years -- 10 Months
Bed : 2

Helpful Information For X-Ray:

Bronchial asthmal : No   Diabetes : No   First day of last menstrual period :
Pregnancy : Yes   S.Creatinine : 0

Specific Reason (s) for Request :

PG, 27WKS, RT LOIN PAIN

Case View :

PG, 27WKS, RT LOIN PAIN

Examination Required :

MR Pelvis

*Signature & Stamp
Dr. RAGHAD SAMI BO GHAIS

Report :   Date : 13-02-2025

MR Pelvis of 13-FEB-2025:
History:
A 27wks pregnant female with right loin pain , was diagnosed as pyelonephritis however clinically it is suspected to have appendicitis.

Findings:

??Encysted fluid collection is located in the right iliac fossa just below the cecum pole and inseparable from the right adnexa. It measures 1.9 x 3.8 cm in max. cross sectional diameters , displaying high T2 and low T1 signals with no significant bright signal in DWI. Minimal nearby free fluid signal is also noted.
??Inadequately distended tubular structure is seen close to the medial wall of the ascending colon? collapsed appendix with clear surrounding fat planes and no radiological evidence of regional inflammatory changes could be appreciated. No regional lymph nodes could be demonstrated.
??Gravid uterus is extending up to the abdominal region and displacing the surrounding bowel loops laterally. It contains single moving fetus with left posteriolaterally located placenta seen not obliterating the cervical os.
??Normal appearance of the rectum and ischiorectal fossa on both sides.
??Smooth filling of the urinary bladder with no mural thickening or diverticular out pouching.
??Normal appearance of the other pelvic organs.
??Normal marrow signal pattern of the pelvic bones.

Conclusion:
??Right iliac fossa encysted fluid collection , likely adnexal in origin , kindly compare with U/S results.
??Suspected collapsed appendix with no radiological evidence of active inflammatory changes could be appreciated , for clinical and laboratory correlation.

* This document is digitally signed, No need for Dr. Signature.   * هذه الوثيقة موقعة إليكترونيا ولا تحتاج إلى توقيع من الطبيب.

Scanned with CamScanner

LABORATORY REPORT

Accession No: 2502165510

FARWANIYA HOSPITAL - KUWAIT.
SUBAH AL-NASER,BLOCK 6, BLDG NO. 900016.,
P.O.BOX NO. 18373, CODE NO. 81004,
STATE OF KUWAIT
Tel: 24802286, FAX: 24893078.

PATIENT

AYA HASSAN IBRAHIM
Hosp ID:**F1048838**
Date of Birth:**28/10/1999**
Location:**Obs & Gyne Emergency NF2** Room No: U
Ordered On:**12/02/2025 23:55:32**

Civil ID:**299102803081**
Passport No:
Gender: **Female**
Bed no:U
Req no:**8961164**

Ordered By... Dr.HUSAM HASSAN ZELZELAH

CLINICAL CHEMISTRY

Test Description	Result Value			Reference Range	Unit
**Profile Name : Liver Profile (LFT)- (Alb, Tbil, ALP, ALT), Kidney Profile (RFT)- (Urea, Crea, Na, K, Cl, CO2)**					
**Sample No:2502181064**					
eGFR (Calculated)	153				
Urea	**2.3**	**L**	⇩	**2.8-7.2**	**mmol/L**
Creat	**30**	**L**	⇩	**49-90**	**umol/L**
Sodium	**135**	**L**	⇩	**136-146**	**mmol/L**
Potassium	3.8			3.5-5.1	mmol/L
Chloride	104			101-109	mmol/L
Bicarbonate	**20**	**L**	⇩	**21-31**	**mmol/L**
Albumin	**34**	**L**	⇩	**35-52**	**g/L**
Total Protein	**64**	**L**	⇩	**66-83**	**g/L**
Uric acid	192			155-357	umol/L
Total Bilirubin	5			5-21	umol/L
Alk. Phos	**114**	**H**	⇧	**33-98**	**IU/L**
ALT	34			3-35	IU/L
Anion Gap	14.8				mmol/L

Released By: **EIDA ALENZI on 13/02/202500:35:42**

Special Range:

Component	Sex	Ref Range
eGFR (Calculated)	FEMALE	CKD Stage1 >90 mL/min<br>CKD Stage2 60-89 mL/min<br>CKD Stage3 30-59 mL/min<br>CKD Stage4 15-29 mL/min<br>CKD Stage5 <15 mL/min<br>If Patient >=70 yrs egfr should be interpreted with caution.
eGFR (Calculated)*	FEMALE	CKD Stage1 >90 mL/min<br>CKD Stage2 60-89 mL/min<br>CKD Stage3 30-59 mL/min<br>CKD Stage4 15-29 mL/min<br>CKD Stage5 <15 mL/min<br>If Patient >=70 yrs egfr should be interpreted with caution.

Printed By : **EIDA ALENZI** Date:**13/02/2025** Time:**00:35:45**

Scanned with CamScanner

LABORATORY REPORT

Accession No: 25021656[illegible]

FARWANIYA HOSPITAL - KUWAIT.
SUBAH AL-NASER,BLOCK 6, BLDG NO. 900016,,
P.O.BOX NO. 18373, CODE NO. 81004,
STATE OF KUWAIT
Tel: 24802286, FAX: 24893078.

PATIENT

AYA HASSAN IBRAHIM
Hosp ID:F1048838
Date of Birth:28/10/1999
Location:Obs & Gyne Emergency NF2 Room No: U
Ordered On:13/02/2025 01:47:34

Civil ID:299102803[illegible]
Passport No:
Gender: Female
Bed no:U
Req no:8961164

Ordered By ...Dr.,HUSAM HASSAN ZELZELAH

MICROBIOLOGY

Sample No:2502181250

Testname	Results
	**Urine - routine , microscopy**
**BLD**	: neg /ul
**PRO**	: 0.15 g/L
**GLU**	: norm mmol/L
**Colour**	: Yellow
**Clarity**	: Light turbid
**SG**	: 1.030
**PH**	: 5
**KET**	: 15 mmol/L
**NIT**	: neg
**BIL**	: neg umol/L
**UBG**	: norm umol/L
**LEU**	: 500 /ul
**RBC**	: 0-5
**WBC**	: >50
**Yeast Cells**	: -
**Granular Cast**	: NIL
**RBC Cast**	: NIL
**WBC Cast**	: NIL
**Waxy Cast**	: NIL
**Uric Acid Crystals**	: NIL
**Calcium Oxalate Monohydrate**	: NIL
**Calcium Oxalate Dihydrate**	: NIL
**Triple Phosphate**	: NIL
**Uric Acid**	: NIL
**Calcium Phosphate**	: NIL
**Pathologic Cast**	: NIL

Released By: **ANNIE SHINE on 13/02/2025 1:53:57 AM 01:53:57**

Printed By : **ANNIE SHINE** Date:**13/02/2025** Time:**01:54:00**

End Of Report

Scanned with CamScanner

LABORATORY REPORT

Accession No: 2502171337

FARWANIYA HOSPITAL - KUWAIT.
SUBAH AL-NASER,BLOCK 6, BLDG NO. 900016,,
P.O.BOX NO. 18373, CODE NO. 81004, STATE OF KUWAIT
Tel: 24802286, FAX: 24893078.

PATIENT	Inpatient
**AYA HASSAN IBRAHIM**	Civil ID:**299102803081**
Hosp ID:**F1048838**	Passport No:
Date of Birth:**28/10/1999**	Gender: **Female**
Location:**Gyne & Post Natal High Risk C5 NF2** Room No: **7**	Bed no:**1**
Ordered On:**14/02/2025 10:23:38**	Req no:**8961508**

Ordered By... Dr.Amany Elsayed Mosad Elsayed Younis

CLINICAL CHEMISTRY

Test Description	Result Value	Reference Range	Unit
**Sample No:2502187423**			
Amylase	76	28-100	U/L
Released By: **Shiva S. on 14/02/202511:13:16**			

Printed By : **Shiva S.** Date:**14/02/2025** Time:**11:13:19**

Scanned with CamScanner

LABORATORY REPORT

Accession No: 2502171338

FARWANIYA HOSPITAL - KUWAIT.
SUBAH AL-NASER,BLOCK 6, BLDG NO. 900016,,
P.O.BOX NO. 18373, CODE NO. 81004,
STATE OF KUWAIT
Tel: 24802286, FAX: 24893078.

PATIENT	Inpatient
AYA HASSAN IBRAHIM	Civil ID:299102803081
Hosp ID:F1048838	Passport No:
Date of Birth:28/10/1999	Gender: Female
Location:Gyne & Post Natal High Risk C5 NF2 Room No: 7	Bed no:1
Ordered On:14/02/2025 10:23:42	Req no:8961508

Ordered By... Dr.Amany Elsayed Mosad Elsayed Younis

CLINICAL CHEMISTRY

Test Description	Result Value	Reference Range	Unit
**Sample No:2502187424**			
LIPASE	44	11-51	U/L
Released By: Shiva S. on 14/02/202511:13:35			

Printed By : **Shiva S.** Date:**14/02/2025** Time:**11:13:40**

Scanned with CamScanner

LABORATORY REPORT

Accession No: 2502171219

FARWANIYA HOSPITAL - KUWAIT.
SUBAH AL-NASER,BLOCK 6, BLDG NO. 900016,,
P.O.BOX NO. 18373, CODE NO. 81004,
STATE OF KUWAIT
Tel: 24802286, FAX: 24893078.

PATIENT	Inpatient
**AYA HASSAN IBRAHIM**	Civil ID:**299102803081**
Hosp ID:**F1048838**	Passport No:
Date of Birth:**28/10/1999**	Gender: **Female**
Location:**Gyne & Post Natal High Risk C5 NF2** Room No: **7**	Bed no:**1**
Ordered On:**14/02/2025 09:27:53**	Req no:**8961508**

Ordered By... Dr.Amany Elsayed Mosad Elsayed Younis

CLINICAL CHEMISTRY

Test Description	Result Value	Reference Range	Unit
**Sample No:2502187304**			
Lactate	0.88	0.5-2.2	mmol/L
Released By: **Shiva S. on 14/02/202511:37:17**			

Printed By : **Shiva S.** Date:**14/02/2025** Time:**11:37:19**

Scanned with CamScanner

LABORATORY REPORT

Accession No: 2502171311

FARWANIYA HOSPITAL - KUWAIT.
SUBAH AL-NASER,BLOCK 6, BLDG NO. 900016,,
P.O.BOX NO. 18373, CODE NO. 81004,
STATE OF KUWAIT
Tel: 24802286, FAX: 24893078.

PATIENT	Inpatient
**AYA HASSAN IBRAHIM**	Civil ID:**299102803081**
Hosp ID:**F1048838**	Passport No:
Date of Birth:**28/10/1999**	Gender: **Female**
Location:**Gyne & Post Natal High Risk C5 NF2** Room No: **7**	Bed no:**1**
Ordered On:**14/02/2025 10:17:08**	Req no:**8963396**

Ordered By... Dr.AISHA RAFIQUE

HEMATOLOGY

Test Description	Result Value			Reference Range	Unit
**Sample No:2502187397**					
WBC	**12.0**	**H**	⇧	**3.9-11.1**	**10^9/L**
Neutrophils%	**75.5**	**H**	⇧	**39.9-73**	**%**
Lymphocytes%	**18.4**	**L**	⇩	**18.8-50.8**	**%**
Monocytes%	5.5			0-12.2	%
Eosinophils%	0.3			0-6	%
Basophils%	0.3			0-1.8	%
NRBC%	0.1			0-1	%
Neutrophils#	**9.1**	**H**	⇧	**1.8-7.1**	**10^9/L**
Lymphocytes#	2.2			1.2-3.6	10^9/L
Monocytes#	0.7			0-2	10^9/L
Eosinophils#	**0.0**	**L**	⇩	**0.1-0.5**	**10^9/L**
Basophils#	0.0			0-0.3	10^9/L
NRBC#	0.0				10^9/L
RBC	**3.18**	**L**	⇩	**3.88-5.2**	**10^{12}/L**
Hb	**87**	**L**	⇩	**118-148**	**g/L**
Hct	**0.265**	**L**	⇩	**0.36-0.44**	**L/L**
MCV	83.3			82-98	fL
MCH	27.4			27.3-32.6	pg
MCHC	328			316-370	g/L
RDW	12.9			11.6-15.5	%
Plt	277			150-450	10^9/L
MPV	11.1			6.5-11.6	fL
IG#	**0.21**	**H**	⇧	**0.01-0.03**	
IG%	**1.70**	**H**	⇧	**0-1**	**%**

Released By: **Aryam . on 14/02/202510:37:05**

Printed By : **Aryam .** Date:**14/02/2025** Time:**10:37:07**

Scanned with CamScanner

Ministry Of Health
Farwaniya Hospital

Operation Sheet

Main Theatre

File No F1048838

Ward	
Patient Name	ايه حسن ابراهيم صبره
Sex	أنثى
Age	26
Doc. in Charge	
Surgeon	AHMAD MOHAMMAD ABDULTAWAB ALSHARAIBNI, Taher Abdelaziz Hosain Elsharnoby
Anaesthetist	
Nurse	
Pre. Op. Ds :	PG, 27WKS, PERSISTANT RT LOIN PAIN
Post Op. Ds :	PG, 27WKS, PERSISTANT RT LOIN PAIN
Operation Name	Salpingotomy
Operation date :	
Starting Time :	19:15
Finishing Time :	20:00

Operative procedure (inclunding finding, the codition of all examined, and procedures incision, ligatures, sutures, drainage, swab count, and closure):

Under Ga Sterilization & Dropping
Tractocele Bolus Given
Trocar 10 At Pulmares Point Inserted After Gas Inflation
Panaramic View Ther Wase Gangerenous Rt Tube Twistwd 6 Times
2 Trocar Inserted Under Vision 1 Right & 1 Left
Tube Coagulated And Cuted Below The Site Of Tortion
Sergeone Came Appendix Normal & No Other Abnormality
Rt Tube Sent For H/P
U/S Done Viable Fetus

Transfer To Lr For 6 Hs
Trctocele Protocol For 24 Hs
Dexamethason Protocol
Rocephin 2 G Od
Pethedin 100 Mg Sos
Perphalgan Ivd Qds

Specimen(s)to Pathology :

Swab count correct sign. Yes

Postoperative Instruction, Next page

Scanned with CamScanner
